# Electroacupuncture for tapering off long-term benzodiazepine use: study protocol of randomized controlled trial

**DOI:** 10.1186/s12906-017-1692-5

**Published:** 2017-03-31

**Authors:** Wing-Fai Yeung, Ka-Fai Chung, Zhang-Jin Zhang, Wai-Chi Chan, Shi-Ping Zhang, Roger Man-Kin Ng, Connie Lai-Wah Chan, Lai-Ming Ho, Yee-Man Yu, Li-Xing Lao

**Affiliations:** 1 The Hong Kong Polytechnic University, Hunghom, Kowloon, Hong Kong SAR China; 2 Department of Psychiatry, University of Hong Kong, Pokfulam, Hong Kong SAR China; 3 School of Chinese Medicine, University of Hong Kong, Pokfulam, Hong Kong SAR China; 4 School of Chinese Medicine, Hong Kong Baptist University, Kowloon Tong, Kowloon, Hong Kong SAR China; 5 Department of Psychiatry, Kowloon Hospital, 147A Argyle Street, Kowloon, Hong Kong SAR China; 6 Department of Psychiatry, United Christian Hospital, 130 Hip Wo Street, Kwun Tong, Kowloon, Hong Kong SAR China; 7 School of Public Health, University of Hong Kong, Pokfulam, Hong Kong SAR China

**Keywords:** Benzodiazepine discontinuation, Withdrawal, Acupuncture, Sham, RCT

## Abstract

**Background:**

Conventional approaches for benzodiazepine tapering have their limitations. Anecdotal studies have shown that acupuncture is a potential treatment for facilitating successful benzodiazepine tapering. As of today, there was no randomized controlled trial examining its efficacy and safety. The purpose of the study is to evaluate the efficacy of using electroacupuncture as an adjunct treatment to gradual tapering of benzodiazepine doses in complete benzodiazepine cessation in long-term benzodiazepine users.

**Methods/Design:**

The study protocol of a randomized, assessor- and subject-blinded, controlled trial is presented. One hundred and forty-four patients with histories of using benzodiazepines in ≥50% of days for more than 3 months will be randomly assigned in a 1:1 ratio to receive either electroacupuncture or placebo electroacupuncture combined with gradual benzodiazepine tapering schedule. Both experimental and placebo treatments will be delivered twice per week for 4 weeks. Major assessments will be conducted at baseline, week 6 and week 16 post-randomization. Primary outcome is the cessation rate of benzodiazepine use. Secondary outcomes include the percentage change in the doses of benzodiazepine usage and the severity of withdrawal symptoms experienced based on the Benzodiazepine Withdrawal Symptom Questionnaire, insomnia as measured by the Insomnia Severity Index, and anxiety and depressive symptoms as evaluated by the Hospital Anxiety and Depression Scale. Adverse events will also be measured at each study visit.

**Discussion:**

Results of this study will provide high quality evidence of the efficacy and safety of electroacupuncture as an adjunct treatment for benzodiazepine tapering in long-term users.

**Trial registration:**

ClinicalTrials.gov NCT02475538.

**Electronic supplementary material:**

The online version of this article (doi:10.1186/s12906-017-1692-5) contains supplementary material, which is available to authorized users.

## Background

Benzodiazepines are commonly prescribed for short-term relief of anxiety and insomnia symptoms. Despite the initial intention, some patients continue taking the drugs and become long-term benzodiazepine users. Cross-sectional studies indicated that 58–84% of benzodiazepine users reported taking the drugs for longer than 6 months [1, 2]. In a population-based survey in Switzerland, nearly one-tenth of 520,000 participants reported at least one benzodiazepine use in the last 6 months and among the benzodiazepine users, 56% were taking the drug for more than 90 days [3]. The potential harms due to long-term benzodiazepine use, including abuse, dependence, overdose, cognitive impairment, household, work and road accidents, and falls, often outweigh its benefits [4–6]. Particularly in the elderly, benzodiazepine use, both short and long term, has been associated with increased risk of daytime drowsiness, accidents and falls, hip fractures and mortality [5]. Studies have shown that a high proportion of long-term benzodiazepine users have attempted to stop or reduce taking the drug; however, many of them have failed due to benzodiazepine withdrawal symptoms [7].

Benzodiazepine withdrawal symptoms, such as insomnia, anxiety, hand tremor, sweating, muscle pain and irritability are common [8]. A study showed that 43% of 180 participants who were taking diazepam for longer than 8 months experienced withdrawal symptoms on cessation of use [9]. Another study indicated that 35% of 109 patients with panic disorder reported withdrawal symptoms when an 8-week course of alprazolam was stopped [10]. Gradual reduction or in combination with substitutive pharmacotherapy or psychological intervention are conventional approaches for tapering benzodiazepines [11, 12]. According to a meta-analysis including both adults and older adults, the average cessation rate was 42% for gradual benzodiazepine reduction in routine care. There were no significant benefits for gradual reduction in combination with substitutive pharmacotherapy [11]; however, combining gradual reduction with psychological intervention was more effective than gradual reduction alone (OR = 1.82, 95% CI = 1.25–2.67) [11]. Psychological intervention may have helped patients to attain motivation and confidence or have reduced their levels of anxiety and insomnia during benzodiazepine withdrawal [13]. While the existing conventional tapering approaches need further trials to confirm their effectiveness, exploring complementary and alternative therapies is therefore suggested.

Complementary and alternative medicine is a group of diverse medical and healthcare practices that are not presently considered to be a part of conventional medicine. A recent systematic review showed that the 12-month prevalence of complementary and alternative medicine use ranged between 9.8 and 76% [14]. Complementary and alternative medicine therapies can be an alternative treatment to patients than substitutive pharmacotherapy or psychological intervention as treatments of benzodiazepine tapering. Acupuncture is one of the commonly-used complementary and alternative medicine therapies. According to the traditional Chinese medicine theory, fine needles are inserted at special points on the body, called acupoints, to produce therapeutic effects [15]. Electroacupuncture is a special technique of acupuncture. Instead of manual stimulation, electricity is used to stimulate acupoints via inserted acupuncture needles. Previous systematic reviews have shown that acupuncture is efficacious in alleviating anxiety symptoms in subjects with anxiety [16] and in improving sleep quality in subjects with a chief complaint of insomnia [17, 18]. Anecdotal reports have been performed to examine whether acupuncture can augment gradual benzodiazepine tapering in enhancing benzodiazepine cessation rate [19–21]. Ruan et al. showed that the use of hypnotics was reduced from 7.6 times per week to 3.2 times per week after a 2-month course of electroacupuncture in 32 subjects with insomnia and hypnotic dependence [19]. In another study, the cessation rate of hypnotics was over 90% after 10 sessions of manual acupuncture [20]. Another study showed that all subjects could stop using their benzodiazepines for anxiety following a 2-month manual acupuncture treatment and 80% of the subjects attained a Hamilton Anxiety Rating Scale score below 14 [21]. However, these studies adopted a retrospective recall on the use of benzodiazepine, which is vulnerable to recall bias and the reliability is limited. Besides these uncontrolled studies, to the best of our knowledge, there has been no randomized placebo-controlled study on the efficacy and safety of electroacupuncture as an adjunct treatment of gradual benzodiazepine withdrawal in enhancing benzodiazepine cessation rate. We therefore planned to conduct a randomized controlled trial to examine the short- (2-week posttreatment) and medium-term (12-week posttreatment) effects of electroacupuncture in a group of long-term benzodiazepine users in Hong Kong.

## Methods

### Objective

This study aims to examine the efficacy and safety of electroacupuncture as an adjunct treatment to gradual benzodiazepine withdrawal in enhancing benzodiazepine cessation rate in long-term (at least 3 months) benzodiazepine users. The short-term and medium-term effects of electroacupuncture were defined as within 4 weeks and 4–12 weeks after completion of the electroacupuncture treatment course. We hypothesize that subjects receiving electroacupuncture will have a higher benzodiazepine cessation rate than those receiving non-invasive placebo acupuncture at 2-week posttreatment (short-term, week 6 post-randomization) and 12-week posttreatment (medium-term, week 16 post-randomization).

### Trial design

This study is a randomized, parallel-group, assessor- and subject-blinded controlled trial with a 1:1 ratio of group allocation to receive electroacupuncture plus gradual tapering of benzodiazepines or placebo electroacupuncture plus gradual tapering. Design and reporting of the study will follow the CONSORT [22] and STRICTA [23] recommendations.

### Ethical approval

The study will be conducted in compliance with local law, Declaration of Helsinki (1989), institutional policies and the Good Clinical Practice (ICH-GCP) guidelines to protect subjects’ right and safety. Ethics approval has been obtained from the Institutional Review Board of the University of Hong Kong/Hospital Authority Hong Kong West Cluster (UW 14–554), Research Ethics Committee of Hospital Authority Kowloon Central/Kowloon East Cluster (KC/KE-15-0178/FR-3) and Human Subjects Ethics Sub-committee of the Hong Kong Polytechnic University (HSEARS20160509002). The trial has been registered at ClinicalTrials.gov (NCT02475538).

### Participants

A total of 144 subjects who are long-term benzodiazepine users will be recruited from psychiatric outpatient clinics of three regional hospitals in Hong Kong and an integrative health clinic. We have planned an 18-month recruitment period starting from July 2015.

#### Inclusion criteria

Subjects will be included if they are: (1) 18 years or above in age including elderly patients; (2) having at least one of the psychiatric diagnoses that are listed in Table 1; (3) taking one or more benzodiazepines, coded as N05BA, N05CD, N05CF, and M03BX07 according to the World Health Organization Anatomical Therapeutic Chemical classification system [24], on more than 50% of days for at least 3 months and during a prospective 2-week period prior to baseline; and (4) willing to taper benzodiazepines as per protocol.

**Table 1:** Lists of included and excluded psychiatric disorders according to the ICD-10 system

Included	
F32.0 Mild depressive episode; F32.1 Moderate depressive episode; F32.8 Other depressive episodes; F32.9 Depressive episode, unspecified; F33.0 Recurrent depressive disorder, current episode mild; F33.4 Recurrent depressive disorder, currently in remission; F33.1 Recurrent depressive disorder, current episode moderate; F33.8 Other recurrent depressive disorders; F33.9 Recurrent depressive disorder, unspecified; F41.0 Panic disorder; F41.1 Generalized anxiety disorder; F41.2 Mixed anxiety and depressive disorder; F43.2 Adjustment disorders; F51.0 Nonorganic insomnia	
Excluded	
F31.0 Bipolar affective disorder; F42.0 Obsessive-compulsive disorder; F43.1 Post-traumatic stress disorder; F20.0 Schizophrenia; F21–29 other Schizotypal and delusional disorders; F55.0 Abuse of non-dependence-producing substances; F10–12, F14–19 Abuse of other psychoactive substances	

#### Exclusion criteria

We will exclude participants who have: (1) any increase by 50% or higher in the dosage of antidepressants or anxiolytics in the past 1 year; (2) scored ≥8 in either the depression or anxiety subscale of the Hospital Anxiety and Depression Scale (HADS) [25]; (3) any concurrent psychiatric disorders on the exclusion list (Table 1); (4) any unstable psychiatric conditions or serious physical illnesses which are judged by the investigator to render unsuitable or unsafe to join the study; (5) valvular heart defects or bleeding disorders, taking anticoagulant drugs, or are fitted with any implanted electrical device such as pacemaker, defibrillator, or brain stimulation; (6) acupuncture during the previous 6 months prior to baseline; (7) pregnancy, breastfeeding or childbearing potential but not using adequate contraception; (8) infection or abscess close to the site of selected acupoints and in the investigator’s opinion inclusion is unsafe; and (9) significant suicidal risk as rated by the Hamilton Depression Rating Scale (HDRS) [26] item on suicide (a score ≥ 3).

### Trial procedure (Fig. 1)

Potential subjects will be invited to attend a face-to-face interview for written consent, psychiatric history, and history of benzodiazepine use. We will request participants to complete a daily record of benzodiazepine use in the 2 weeks prior to baseline. Those who use benzodiazepines for more than 50% of days are eligible for randomization. Participants will be randomly assigned to receive electroacupuncture plus gradual tapering or placebo electroacupuncture plus gradual tapering in a 1:1 ratio. Block randomization will be administrated by an independent administrator using a computer-generated list of random sequence. Group allocation will be kept in sequentially-numbered opaque-sealed envelopes and will be opened by acupuncturists after all baseline assessments have been completed by the research assistant who is blind to treatment allocation.

**Fig. 1:**
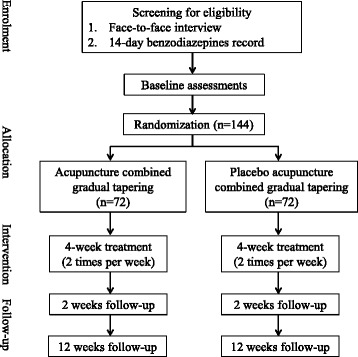
Flowchart of study procedures

### Intervention

Electroacupuncture combined with gradual tapering.

Subjects will receive electroacupuncture twice per week for 4 consecutive weeks. The frequency and duration are based on previous systematic reviews [16–18] and experts’ opinions. Subjects will be needled at bilateral EX-HN1 (Sishencong), EX-HN22 (Anmian), GB8 (Shuaigu), ST8 (Touwei), EX-HN5 (Taiyang), GB15 (Toulinqi), PC6 (Neiguan), HT7 (Shenmen), SP6 (Sanyinjiao), LV3 (Taichong), unilateral EX-HN3 (Yintang), GV24 (Shenting), and GV20 (Baihui). The location and indication are summarized in Table 2. The total number of needles used in each session will be fixed to 23. The acupoints have been used for treating insomnia and mood disorders [16, 27–29] and modified for the purpose of benzodiazepine tapering according to our expert team.

**Table 2:** Location and indication of acupoints used in the treatment protocol

Acupoints	Location	Indication in Traditional Chinese Medicine
EX-HN1 (Sishencong)	At the vertex of the scalp, four points, 1 *cun* respectively anterior, posterior and lateral to GV 20 (baihui)	Tranquilize and calm the mind, helps in headache, insomnia and forgetfulness
EX-HN22 (Anmian)	Midpoint between SJ 17 and GB 20	Helps in insomnia, palpitations and restlessness
GB8 (Shuaigu)	Head, directly above auricular apex, 1.5 *cun* superior to the hairline	Clear haet and extinguish wind, helps in headache and dizziness
ST8 (Touwei)	On the head 0.5 *cun* directly superior to anterior hairline, at the corner of forehead, 4.5 *cun* lateral to the anterior median line	Clear the head, helps in headache, dizziness and eye pain
EX-HN5 (Taiyang)	At the temple in the depression about one finger-breadth posterior to the midpoint of the lateral end of the eyebrow and the outer canthus	Helps in mental disorders such as headache, insomnia, forgetfulness, epilepsy and eye disorders
GB15 (Toulinqi)	On the head 0.5 *cun* within the anterior hairline, directly superior to the center of the pupil	Calm the mind, helps in headache, dizziness, double vision and tinnitus
PC6 (Neiguan)	On the medial aspect of the forearm between the palmers longus and flexor carpi radials tendons, 2 *cun* proximal to the palmers wrist crease	Calm the heart and mind, helps in palpitations, vexation, insomnia, depression, mania and other heart and mind disorders
HT7 (Shenmen)	Posteromedial aspect of the wrist radial to the flexor carpi ulnaris tendon at the palmer wrist crease	Calm the heart and mind, helps in insomnia, depression, mania, forgetfulness, headache and dizziness
SP6 (Sanyinjiao)	On the tibial aspect of leg posterior to the medial border of the tibia, 3 *cun* superior to the prominence of the medial mallcolus	Helps in insomnia and mania
LV3 (Taichong)	Dorsum of foot, within the depression distal between first and second metatarsal bones.	Calm the liver and extinguish win, helps in depressive psychosis, manic psychosis and insomnia.
EX-HN3 (Yintang)	At the midpoint between the medial ends of the eyebrows	Clear liver heat and improve vision
GV24 (Shenting)	One the head at the anterior median line, 0.5 *cun* superior to the anterior hairline	Subdue yang and calm the mine, helps in depression, mania, insomnia, other mental disorders, headache and dizziness
GV20 (Baihui)	On the head at the anterior median line, 5 *cun* superior to the anterior hairline	Helps in palpitations due to fright, insomnia, forgetfulness and other mental disorders

After sterilizing the skin around the acupoints with 75% alcohol, sterilized disposable needles (Blister Needle, Dong Bang, Korean, 0.25x30mm) will be inserted. “De qi” (a radiating feeling of numbness or distension considered to be indicative of effective needling as reported by the participant) will be achieved if possible. It is an indication of “effective needling” in terms of TCM theory. Four pairs of needles (left and right EX-HN1; GV20-EX-HN3; left GB8- ST8; and right GB8- ST8) will be connected to an electric-stimulator (AWQ 104 L, Hong Kong) for continuous stimulation at constant current and a frequency of 4 Hz. In clinical practice, acupuncturists usually select 1–4 pairs of acupoints to deliver electric stimulation. These four pairs of acupoints were chosen to deliver electric-stimulation because they are the main acupoints that are supposed to have anxiolytic and sedative effects according to the TCM theory. The amplitude of electrical stimulation will be adjusted to a comfortable level. The needles will be left for 30 min and then removed which resembles the duration used in clinical practice.

**Fig. 2:**
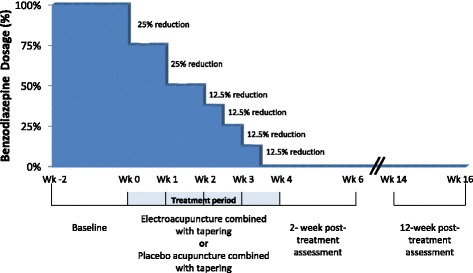
Benzodiazepine gradual tapering schedule

All the included subjects will be advised to taper their benzodiazepines over 4 weeks according to a protocol as suggested by Rickels et al. [30]. The baseline benzodiazepine dosage in diazepam equivalent will be calculated based on the average daily consumption in the 2 weeks prior to baseline [31]. Subjects will be asked to reduce their daily dose by 25% in the first and second week. For the remaining 50%, we will advise reduction by 12.5% for 3–4 days each time (Fig. 2). Where tablets do not allow precise dose reduction, tablets will be either cut in half or spaced over alternative days as required. After the second, fourth, sixth, and eighth electroacupuncture sessions, the subjects will be asked to complete a Benzodiazepine Withdrawal Symptom Questionnaire [32] to assess their withdrawal symptoms, then a trained research assistant who is blinded to the treatment allocation will use about 10–15 min to evaluate subjects’ benzodiazepine withdrawal symptoms and other adverse events, discuss the problems encountered due to benzodiazepine tapering, count the surplus of benzodiazepines due to dose reduction, set the withdrawal schedule for the following weeks, and provide support and encouragement to follow the withdrawal schedule. If subjects find it too difficult to cope, feel unable to meet the reduction goal, or have at least one item in the Benzodiazepine Withdrawal Symptom Questionnaire [32] rated as severe, we will suggest keeping the dosage unchanged or slowing down the tapering, e.g., the 25% per week tapering in the first 2 weeks can be reduced to 12.5% per week.

#### Placebo electroacupuncture combined with gradual tapering

The sterilization procedure will be the same as in electroacupuncture group. Placebo needles, designed by Streitberger [33], have a blunt tip that cannot penetrate the skin. The handles of placebo needles will slide over the needle when they are pressed, giving an appearance of penetrating the skin. The placebo needles will be placed 1 in. beside the acupoints in order to avoid acupressure effect. The needles are held by surgical tape or hair pin to imitate the procedure of electroacupuncture. The needles are connected to an electric-stimulator with zero frequency and amplitude. The number, duration and frequency of treatment session will be the same as in the electroacupuncture group.

Electroacupuncture will be performed according to a standard operating procedure (SOP) manual which is developed to standardize the treatment procedure and dialogue between acupuncturists and subjects (Additional file 1).

### Fidelity of the intervention

The acupuncturists are registered Chinese medicine practitioners in Hong Kong with a Bachelor degree in Chinese Medicine and at least 5 years’ experience in providing needle acupuncture. Their first 10 electroacupuncture treatments will be assessed and guided by the PI (WY) using a standardized checklist to ensure fidelity of the intervention. The PI will also randomly visit to check the acupuncturists’ adherence to the research protocol.

### Outcome measures (Table 3)

#### Primary outcome

Primary outcome is the proportion of participants who successfully discontinue benzodiazepines at 2-week posttreatment (week 6) and 12-week posttreatment (week 16). Previous randomized controlled trials of benzodiazepine tapering [10] have used cessation rate as the main outcome measure. The primary end-point is 12-week posttreatment (Fig. 2).

**Table 3:** Summary of outcome measures and assessment schedule

Measure	Baseline	Intervention				2-week posttreatment	12-week posttreatment
		2nd	4th	6th	8th		
BZ cessation rate^a^	✓					✓	✓
BZ dose reduction, %	✓	✓	✓	✓	✓	✓	✓
BWSQ	✓	✓	✓	✓	✓	✓	✓
ISI	✓		✓			✓	✓
HADS	✓		✓			✓	✓
SDS	✓						
CTRS	✓	✓			✓		
AE monitoring		✓	✓	✓	✓	✓	✓

*BZ* Benzodiazepine, *BWSQ* Benzodiazepine Withdrawal Symptom Questionnaire, *ISI* Insomnia Severity Index, *HADS* Hospital Anxiety and Depression Scale, *SDS* Substance Dependence Scale, *CTRS* Credibility of Treatment Rating Scale, *AE* adverse events

^a^Primary outcome

#### Secondary outcomes

Secondary measures include percentage benzodiazepine dose reduction, Benzodiazepine Withdrawal Symptom Questionnaire (BWSD), Insomnia Severity Index (ISI), and HADS. A daily dose of benzodiazepine in diazepam equivalent and percentage reduction as compared to baseline will be derived from a 14-day record form. Previous studies have suggested that prospective usage diary is more accurate than retrospective recall of consumption [34, 35]. The subject will be asked to bring back their un-used medications at each visit for cross-checking with the usage record.

Benzodiazepine withdrawal symptoms will be assessed by the 20-item self-administrated BWSQ [32]. The items include main symptoms experienced during withdrawal from benzodiazepine dependent patients including dizziness, pains in muscle, muscle twitching, and so on [32]. Withdrawal symptoms are rated as “absent”, “moderate” or “severe” (0, 1, or 2), yielding a total score ranging between 0 and 40. The original English version has been translated into Chinese for use in this study. The back-translated version has been compared with the original version to ensure it appropriately reflects the original version. Insomnia, one of the most common benzodiazepine withdrawal symptoms, will be further evaluated by the ISI [36]. The ISI is a 7-item 5-point self-rating Likert scale to indicate subjects’ perceived severity of insomnia symptoms. The total score ranges from 0 to 28. The Chinese version of ISI has been demonstrated to have adequate validity and reliability [37]. The HADS [25], a validated and widely-used self-reported questionnaire, will be adopted to measure the severity of depressive and anxiety symptoms. It consists of 14 items; seven of them are on depression and seven on anxiety, which generates the anxiety and depression subscales. Somatic symptoms are not included in the HADS. The validated Chinese version of HADS will be used in the present study [38].

### Treatment expectancy

The Credibility of Treatment Rating Scale (CTRS), a 4-item scale, will be used for assessing subjects’ confidence and expectation towards treatment [39]. The Chinese version CTRS has been adopted in previous studies [27, 29]. Higher scores indicate greater confidence and expectation towards treatment.

### Assessment for substance dependence

The Substance Dependence Scale (SDS) measures the severity of dependency towards a particular substance in the last 12 months [40]. The SDS contains five items and the Chinese version has been shown to be reliable and valid [41]. The term “particular substance” in the SDS will be revised to “benzodiazepine” in this study.

### Safety concern

Subjects will be provided with sufficient information regarding the potential adverse events of benzodiazepine withdrawal. Benzodiazepine withdrawal symptoms, and electroacupuncture-related adverse events will be assessed at each study visit. Benzodiazepine withdrawal symptoms will be assessed by the BWSQ. A standardized electroacupuncture-related adverse event form [42, 43], consisting of eight items on adverse events around the needle sites, 13 items on systemic adverse events, and three items on serious adverse events, will be used. Severity of each adverse event will be rated as “absent”, “mild”, “moderate” or “severe” and the causality of adverse event will be reported using a 5-point Likert scale, ranging from “unrelated” to “certain”. In addition, worsening of pre-existing medical and psychiatric conditions is counted as adverse events. Subjects with heart rate over 100 beats per minute, systolic blood pressure higher than 140 mmHg, diastolic blood pressure higher than 90 mmHg, and any suicidal risk based on the HDRS suicidality item will be evaluated by the Principal Investigator before continuing in the study. Those with moderate suicidal risk will be excluded from the study and referred to their case doctors as early as possible.

### Blinding assessment

As all outcome measures are self-reported, assessments are deemed to be blinded. Success of participant blinding will be measured at the last electroacupuncture session. Subjects will be asked a standard question: “When you volunteered for the trial, you were informed that you would receive traditional acupuncture, or acupuncture-like placebo treatment. Which acupuncture do you think you received?” The standard question is adapted from the paper by Park et al. [44].

### Sample size calculation

Sample size calculation is based on benzodiazepine cessation rate, the primary outcome. No previous studies have been conducted on the efficacy of electroacupuncture and placebo electroacupuncture for tapering benzodiazepines; hence the effect size for sample size calculation is estimated according to previous randomized controlled trials using psychological intervention. A median cessation rate of 74% (range = 13–85%) was reported in a previous systematic review on benzodiazepine tapering using psychological intervention [11]. We estimate that electroacupuncture will produce a cessation rate similar to that of psychological intervention (75%) and the cessation rate in placebo control group is about 50%; hence. a 25% difference in cessation rate between groups is assumed [27]. A power of 80% has been regarded as a reasonable protection against Type II error [45]. Based on a 25% between-group difference and a power of 80%, the minimal number of subjects that are required to avoid a Type I error of 0.05 is 108 (54 per group). The final sample size is 144 (72 per group) after allowing a 25% attrition rate at 12-week posttreatment.

### Statistical analysis

Data will be double-entered and checked for consistency. Data analysis will be performed with an identification code of group A and group B according to a pre-specified statistical analysis plan. The coding of group allocation will be revealed after the completion of data analysis or in case of reports of serious adverse events associated with electroacupuncture or benzodiazepine withdrawal. Between-group difference will be assessed by two-sample t-test or chi-square test at baseline, week 4 (end of treatment), week 6 (2-week posttreatment), and week 16 (12-week posttreatment). The absolute risk reduction and number of subjects that are needed to be treated to obtain one benzodiazepine withdrawal will be estimated at week 16. Attempts will be made for minimizing missing data among subjects who do not return for assessment or have withdrawn from study by mail and phone reminders. Both per protocol and intention-to-treat analyses will be performed. Multiple imputation technique will be used to handle missing values, assuming data are missing at random (MAR). Ten sets of imputed values will be generated to adjust for variability due to imputation, and so ten completed data sets will be created. These completed data sets will be analyzed separately with standard statistical methods, and the results are combined into a single multiple-imputation result [46]. Sensitivity analysis will be conducted to examine the effect of departures from the assumption of MAR on clinical outcomes. The missing data due to dropout cases may be associated with those who rebound in insomnia or anxiety levels, or those who fail to decrease consumption of benzodiazepine. Pattern mixture models with various possible values of the informative missing parameters will be employed to conduct the sensitivity analysis [47]. For dichotomous outcomes, the informative missing parameter specifies the odds ratio between the outcome and missingness indicator. For continuous outcomes, it specifies the mean difference between the unobserved outcome and observed outcome. By varying the informative missing parameter, it is possible to examine the magnitude of departures from MAR assumption on different outcomes. Stata rctmiss will be used to do the sensitivity analysis [48].

### Trial status

Subject recruitment has been started in late July 2015 and is expected to complete in March 2017. Results of the study will be available by the end of 2017. The current study does not include any interim analyses.

## Discussion

Benzodiazepine is one of the most frequently prescribed drugs. Although benzodiazepines play an important role in the treatment of anxiety disorders, insomnia, and physical illnesses such as epilepsy, their use has been questioned due to public concerns about adverse effects and liability to lead to physical dependence and abuse. Despite legislative measures to control the prescription of benzodiazepines [49], long-term benzodiazepine use remains common. Gradual reduction of benzodiazepines with or without substitutive pharmacotherapy or psychological intervention have their limitations, the present study will explore using electroacupuncture as an adjunct for benzodiazepine tapering. In this trial, we aimed to examine the specific effects of electroacupuncture such as needle insertion, *deqi* sensation, and electric-stimulation in tapering off benzodiazepine use. Therefore, we used a non-invasive sham as a control. Our next step is to perform a pragmatic trial on the effectiveness of electroacupuncture compared to other standard interventions (e.g. psychological or pharmacological intervention) for tapering benzodiazepines.

To the best our knowledge, we are the first group to perform a randomized placebo-controlled trial using a well-documented screening process and validated scales to examine the efficacy and safety of electroacupuncture for benzodiazepine tapering. Results of this study will enrich our understanding on the use of electroacupuncture for benzodiazepine cessation.

## Additional file

Additional file 1: The Standardized Operating Procedure (SOP) of Electroacupuncture Treatment. (DOCX 18 kb)

## Abbreviations

AE: Adverse events
BWSQ: Benzodiazepine Withdrawal Symptom Questionnaire
CONSORT: Consolidated Standards of Reporting Trials
CTRS: Credibility of Treatment Rating Scale
DSM-IV (SCID): Diagnostic And Statistical Manual Of Mental Disorders, Fourth Edition (Structured Clinical Interview for DSM Disorders)
HADS: Hospital Anxiety and Depression Scale
ICD: International Classification of Diseases
ICH-GCP: Good Clinical Practice from International Conference on Harmonisation of Technical Requirements for Registration of Pharmaceuticals for Human Use
ISI: Insomnia Severity Index
SDS: Substance Dependence Scale
SOP: Standard operating procedure
STRICTA: Standards for Reporting Interventions in Clinical Trials of Acupuncture
