# Supplementary material for: Electroacupuncture for tapering off long-term benzodiazepine use: study protocol of randomized controlled trial
Source: BMC Complement Altern Med. 2017 Mar 31;17:183. doi: 10.1186/s12906-017-1692-5 (PMC5374579; doi:10.1186/s12906-017-1692-5)
Supplement: Additional file 1: — The Standardized Operating Procedure (SOP) of Electroacupuncture Treatment. (DOCX 18 kb) [file 12906_2017_1692_MOESM1_ESM.docx]

**Additional file 1**

**The Standardized Operating Procedure (SOP) of Electroacupuncture Treatment**

1. Pretreatment introduction

1.1 The Research Assistant will arrange subjects’ first treatment visit and pretreatment assessment. When all the baseline assessment is finished, the acupuncturist will deliver either acupuncture treatment or placebo acupuncture treatment according to the treatment allocation.

1.2 The Acupuncturist will ask:

*1.2.1 For the first treatment*

Acupuncturist “*Nice to meet you! I am XX, I will be responsible for offering you the acupuncture treatment. I will needle at your acupoints on hands and feet. During needling, there may be a little painful, but not too sharp; scalp are full of blood vessels and hence may have a small amount of bleeding after needle removal. It is a normal phenomenon. It is no need to worry too much. Just relax. If you are ready, then please lie in the bed.*”

*1.2.2 For the second to eight treatment*

Acupuncturist “*How are you Mr. / Miss? Is it hot/ cold today?* *If you are ready, then please lie in the bed.*”

1. Sterilizing and needling procedure
   1. Skin around these points will be sterilized by 75% alcohol;
   2. Needle is inserted to each acupoint through a guiding tube;
   3. The Acupuncturist will thrust upward and downward to elicit *deqi;*
   4. The Acupuncturist will ask “*is there any feeling of distension and numbness?*”;
   5. The Acupuncturist should try again if no *deqi* sensation;
   6. A surgical tape (or hair pin on head) is used to hold the needles.
2. Electric-stimulation
   1. The following 4 pairs of acupoints will be connected to the electric-stimulator : bilateral Sishencong (EX-HN1); Baihui (GV20) and Yintang (EX-HN3); left Shuaigu (GB8) and left Touwei (ST8); right Shuaigu (GB8) and right Touwei (ST8);
   2. Electric-stimulation is set to in continuous wave, frequency of 4 Hz, and constant current;
   3. The Acupuncturist will ask “*Is the electric stimulation too strong? Do you have any discomfort?*”;
      1. The acupuncturist will adjust the electric stimulation to subjects’ acceptable level;
      2. In the placebo electroacupuncture group, the electric-stimulator is set to zero amplitude. If subjects ask to increase the stimulation, the acupuncturist will reply “*The electric stimulation is fixed at this level and will not be turned up*”;
   4. Timer is set to 30 min. The acupuncturist will tell the subject “*I will remove the needles in 30 minutes. Please let me know if you have any discomfort during the treatment*”;
3. Removal of needles
   1. The acupuncturist will use a sterilized cotton ball to press the needling site and then remove the needle;
   2. The acupuncturist will check to ensure that all needles are removed;
   3. The needles are put into a sharp box;
   4. The acupuncturist will tell the subject *“This treatment session has been done. You may now get up slowly and we will perform an assessment”;*
   5. The acupuncturist will tell the subject:
      1. For the first to seventh treatment, *“The next treatment session will be on [date]. See you next time, take care!”;*
      2. For the eight treatment, *“You have finished the whole treatment course. Hope you will get well soon!”.*
